# Academic promotion and leadership: ‘moving the needle’ for the enhancement of gender equality in Tunisian higher education institutional members of the RMEI network following the TARGET framework

**DOI:** 10.12688/openreseurope.13217.2

**Published:** 2021-05-13

**Authors:** Monia Chouari, Moncef Ghiss, Anastasia Zabaniotou

**Affiliations:** 1 Faculty of Arts and Human Sciences of Sousse, English Department, University of Sousse, Sousse, Bp.547. Erriadh, 4027, Tunisia; 2 National Engineering School of Sousse, Mechanical Laboratory of Sousse, University of Sousse, Sousse, Bp.264 Erriadh, 4023, Tunisia; 3 Réseau Méditerranéen des Ecoles d’Ingénieurs, Ecole Centrale Marseille, Technopôle de Château Gombert, Marseille, 13451, France; 4 Chemical Engineering Department, Faculty of Engineering, Aristotle University of Thessaloniki, Thessaloniki, Thessaloniki, 54124, Greece

**Keywords:** Academic promotion, Leadership, Higher Education, RMEI network, TARGET project, Tunisia, Mediterranean.

## Abstract

Although job opportunities, recruitment criteria, health insurance and social welfare are equally available regardless of sex, academic promotion at higher education institutions (HEIs) has so far been a challenging issue for women more than men. Even though there are not legislative policies or political strategies proscribing gender discrimination, the under-representation of women in high profile positions is thought-provoking as it was found by this study on collecting segregating data at the Faculty of Arts and Human Sciences of Sousse (FAHSS) and to a lesser degree at the National Engineering School of Sousse (ENISO). Given insufficient research in the area under investigation, and despite the shortage of data needed for examination, this study makes use of and analyses the available data collected from Sousse University. Built upon the findings, this paper sets forth to examine impediments as challenges to progress which are encountered by women. Despite the belief that gender parity has been acquired, it is still a challenge to progress to endorse the culture of gender equality at higher education institutions. The study entails the activities of the gender equality committee created at Sousse University in 2018 with the support of the Mediterranean Network of Engineering Schools (RMEI) and under the framework of the EU TARGET project entitled ‘Taking a reflexive approach to gender equality at Institutional transformation’.

## Introduction

Institutional Gender equality implies that the number of women reaching high profile career in HEIs must be the same as that of men. In line with this definition, access to management opportunities, Research and Innovation project participation and other empowering positions, regardless of gender, have become a must. To achieve these fair objectives, a structural change must be implemented within university culture. As TARGET H2020 project boosts a structural change, we have adopted three dimensions of gender equality as outlined in this collaborative research project. First, the gender balance by examining gender-based issues and investigating the real gap hindering equality. Second, the abolishment of barriers for professional careers development of women by the establishment of Gender Cell at Sousse University and the ENISO’s Center of Equity impacting the different institutions to propagate and disseminate gender norms of equality. Third, the integration of the gender dimension in research content by the establishment of a Master’s Program about Women/Gender Studies as well as the implementation of gender equity and gender equality in different teaching modules at the Faculty of Arts and Human Sciences among other HEIs.

Although the percentage of women at senior levels of academic staff has increased in universities the rate of improvement in the representation of women in higher education institutions (HEIs) is still slow and, above all, women are not achieving full recognition ^1^. Globally, high-level professional profiles and leadership positions in academia have so far been retained by men, despite the increasing number of women teachers and researchers in HEIs. The American Council on Education’s Report in 2016 raises many controversial issues about gender inequality in higher education (HE) including the under-representation of women (approximately 30%) in high-ranking leadership positions, such as the presidency and membership of governing boards ^1, 2^. In response to this gender inequality phenomenon, relevant academic studies have tackled thought-provoking questions that arise in diverse fields in European research centers.

Known as advocates of democracy and human rights, the European Commission scholars have projected a promising plan accentuating the importance of gender equality (GE) in research and innovation (R&I) at the core of the European Research Area (ERA). This incentive prompted European research centers to identify analytical tools to investigate the impediments hindering gender balance in Europe, as well as in countries engaged with European Union (EU) projects, namely the European Institute for Gender Equality (EIGE) ^3^. Of equal importance, the European Commission pursued the calls for delivery of gender equality policies to eliminate sexual discrimination and further enhance women’s empowerment opportunities, especially in the science, technology, engineering and mathematics (STEM) fields, especially in engineering. What mostly strengthens the ERA in this GE debate is the EU Horizon2020 program calls, which aim to enforce the implementation of a gender dimension in all research and innovation as a requisite of project assessment and approval (gender mainstreaming).

Another important issue is sexual harassment in Universities. In 2016, the European Institute for Gender Equality published an inspiring comprehensive study in support of the elimination of sexual distinction, to ensure gender equity and gender equality in academia.

In fact, there have been an increasing number of university male teachers and researchers who have developed their professional career and, systematically, become qualified for deanship electoral campaigns or appointments for positions of responsibility, while their female peers have seldom reached the same high professional profile of professoriate. Therefore, most women fall behind the requirements of the candidacy to reach decision-making positions. With regard to gender imbalance in academic promotion and leadership positions, Pat O’Connor’s (2020) publication on “Multi-Level State Interventions and Gender Equality in Higher Education Institutions: The Irish Case” ^2^ is an outcome of the “Gender, Leadership and Management” laboratory project. What we found inspiring in O’Connor’s article are three focal points: a) the impact of the under-representation of women in the Senior Academic Leadership Enterprise; b) the necessity of cultural change; and c) the possibility of change that arises at the macro level (i.e. the state) before reaching the meso level (i.e. the institution) and micro level (personal).

Despite the substantial importance of such a GE issue at HEIs, in Tunisian academic research there are no key publications in the aforementioned specific area of investigation. Therefore, there is a gap to fill in Tunisian GE research. Even if we assume Tunisian researchers’ interest in GE, their studies have so far been based on literature about Tunisian women and how the Tunisian family law reforms influence their daily life; this is a case study of Grami’s (2008) article “Gender Equality in Tunisia'' ^3^. Another inspiring research is “The Making of Gender Equality in Tunisia and Implications for Development'' (2012) ^4^, which deals with the inheritance issue but remains limited because it does not pay attention to gender inequality in HEIs. A French publication about a report on GE, a preliminary requisite to develop GE in India and three African countries including Tunisia, is important, along withother reports by non-governmental organisations that have been widely published, although these have not tackled the current issue of GE at Tunisian HEIs ^5^. The investigation of academic promotion and leadership issues in tertiary education in Tunisia has not yet been explored as a field of research, while its importance has been highlighted ^6^.

Finally, objectives such as the promotion of gender balance in universities, decision-making, and the sharing of responsibilities (professional, personal and family) have not been fully achieved, and in some Mediterranean countries these are not visible objectives inhibiting women from the full exercise of their legitimate aspirations ^1– 5, 7, 8^.

## Scope and objective of the study

In promotion of better GE, the identification of major issues hindering the implementation of efficient strategies towards institutional change triggered this study.

While the study unveils the established pattern of the deep-seated sexist discrimination at Tunisian HEIs, it aims to investigate gender imbalance and intends to identify the obstructing reasons behind the discrepancies between the percentages of women and men with access to leadership positions which are, most often, interconnected with the academic and professional promotion. At the intersection of both questions, the implementation of context-based and needs-based gender equality strategy (GES) becomes of paramount importance in challenging the prevailing male-dominated stereotypes.

**Figure 1.**
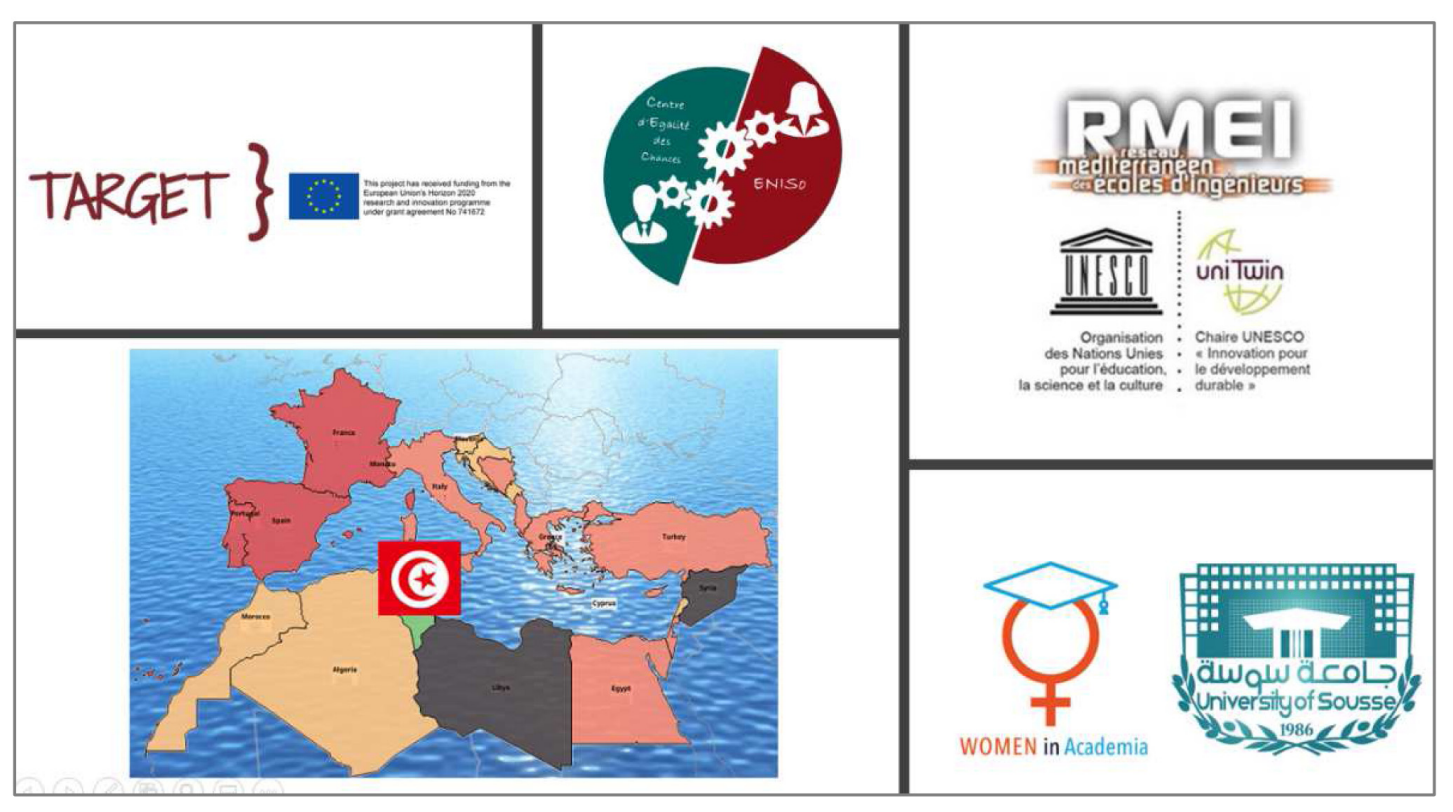
Conceptual representation of the exploration of gender inequality in Tunisian Higher Education Institutional members of the Mediterranean Network of Engineering Schools under the EU TARGET project framework.

Therefore, the current problematic issue about the exploration of gender inequality in the academic promotion in two Tunisian institutions, namely the Faculty of Arts and Human Sciences of Sousse (FAHSS) and National Engineering School of Sousse (ENISO) that is a member of the Mediterranean Network of Engineering Schools (RMEI)benefitted from the EU TARGET project, is a thought-provoking original study in support of the RMEI/TARGET objectives as depicted in the conceptual Figure 1. Following the thread of research in line with the objective of establishing transformation to attain an ideal GE, the conception of this study builds upon GES to endorse and extend on some ideas cited in relevant research papers ^6, 8, 9^.

Therefore, this paper aims to underline the effectiveness of GES that comprise a reflexive vision of conceptualizing GE in two Tunisian HEIs, to provide insights into their application in humanities and STEM contexts and to compare them. 

Such a study is innovative as it draws a comparison between two key areas, humanities and engineering, regarding professional promotion and institutional leadership management.

## Methods

Built upon the aforementioned objective, we designed a mixed research methodology based on segregated data collection and analytical methods that are thematically and structurally applicable, in order to highlight the prominent GE issues at FAHSS and ENISO institutions. We were looking for the exact number of academic staff of both genders, women and men, in different academic grades along with their leadership positions. As raw material, the data was received from Sousse University where the the graphics and tables were based.

With regard to the issue of academic promotion of men and women, the present research covers the period from the time of the recruitment to the present. As to leadership, the period we studied is limited to the last decade (2010–2020), including two disruptions: the post-Arab Spring period and the current year of COVID-19 pandemic, which underpins a burgeoning progress and slight changes in the structure of decision-making positions. The latter is worth highlighting, although the number of women as representatives is less than expected with reference to the recent electoral results, which are clear evidence for the paper’s argument about gender inequality.

We measured male/female inequality in terms of attaining the highest or the penultimate highest academic level in Tunisian HE system of promotion. So, we totaled the number of teachers in each institution before classifying them according to grade A (comprising two categories of full professors and associate professors) and grade B (comprising two categories of assistant and assistant professors). We totaled the number of women and men elected as representatives in institutional scientific councils of 14 institutions at the University of Sousse to elucidate that the findings of the current study about two cases of institutions would be applicable on other institutions or even other Tunisian higher education institutions. This data was collected from Sousse University, and the stastical gender distribution was performed using Excel software.

### Setting place and time

We have started working in the last months of 2020 and we have delayed the accomplishment of the study results waiting for December 2020 final electoral results to measure them in comparison with the previous results confirmation of the study.

### Methodology

This study adopts a mixed method of quantitative and qualitative research to examine the raised topic of promotion and leadership positions either through election and/ or appointment in the case study of two leading institutions: the FAHSS anf the ENISO. We used a simple calculation of percentages of both genders as regards the academic evolution. A post-process of these data using Excel is ensured. A comparison study between FAHSS and ENISO is then illustrated through Excel tables and Histogramms.

To make this study all-inclusive, we included tables and figures for the sake of illustration of the elaboration on the raised controversial issue of gender inequality in HEIs. In this scientific paper, we like to conduct our readers to grasp the significance of gender balance at HEIs straightforwardly by providing them with facts through charts, tables, and diagrams. In line with computational science development, the adopted method of graphics and statistics is a persuasive way to communicate our ideas when words cannot easily articulate them as required, and then back them up by concrete numbers and percentages, facts rather than words. Therefore, readers will only focus on the displayed items like tables, figures and drawings that sum up the main ideas and findings of the study.

### Segregated data collection

Analysis of the available collected data allows us in this pilot study to present statistics and graphs about the academic and professional development of the teaching staff in the key institutions of FAHSS and ENISO, at Sousse University. Data provides us with individual teacher’s information from the date of recruitment to the date of attaining full professor (once attained) going through the gradual promotion process in their individual teaching career.

## Results

**Figure 2.**
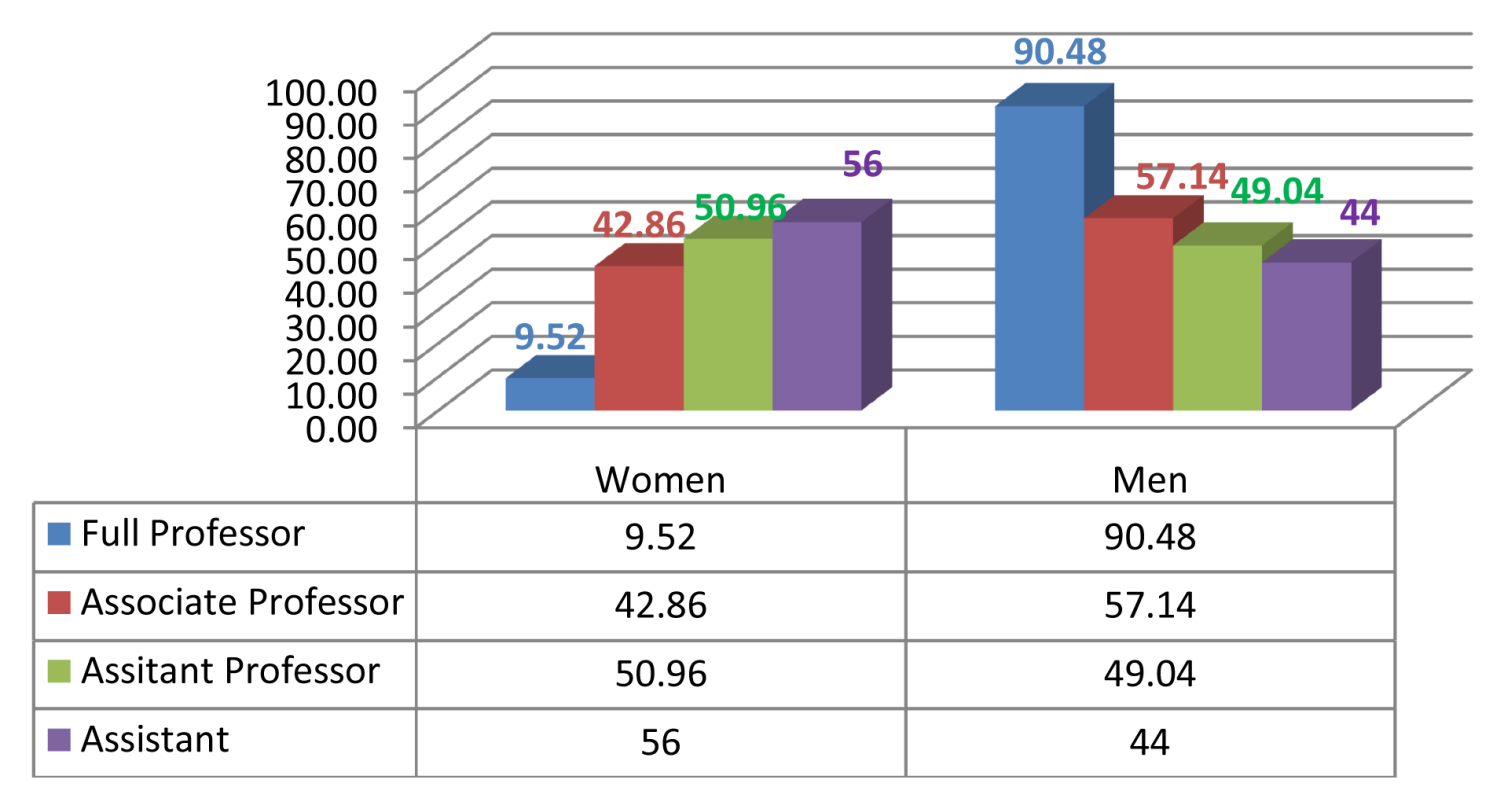
Academic gender evolution at the Faculty of Arts and Human Sciences of Sousse.

**Figure 3.**
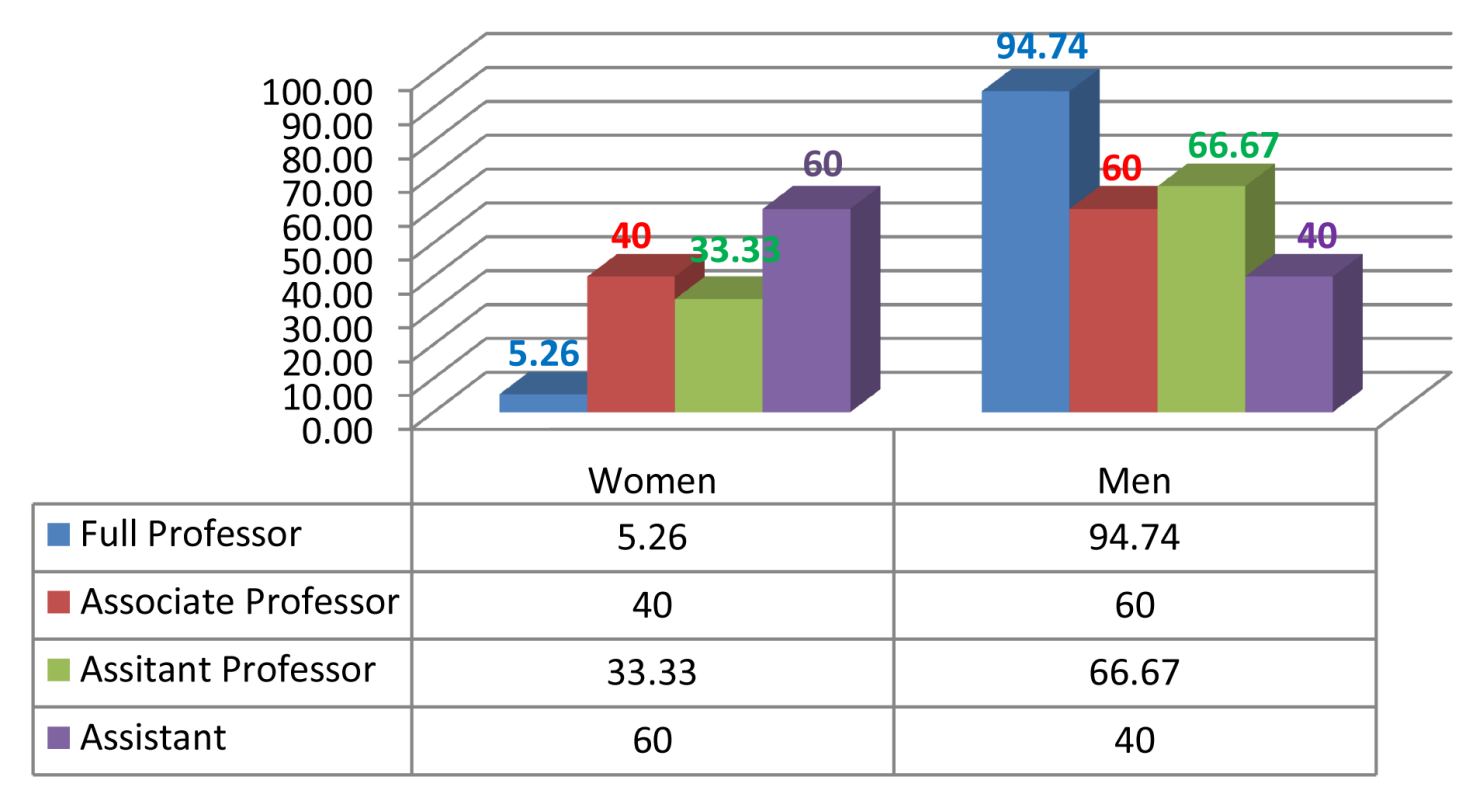
Academic gender evolution at the National Engineering School of Sousse.

**Figure 4.**
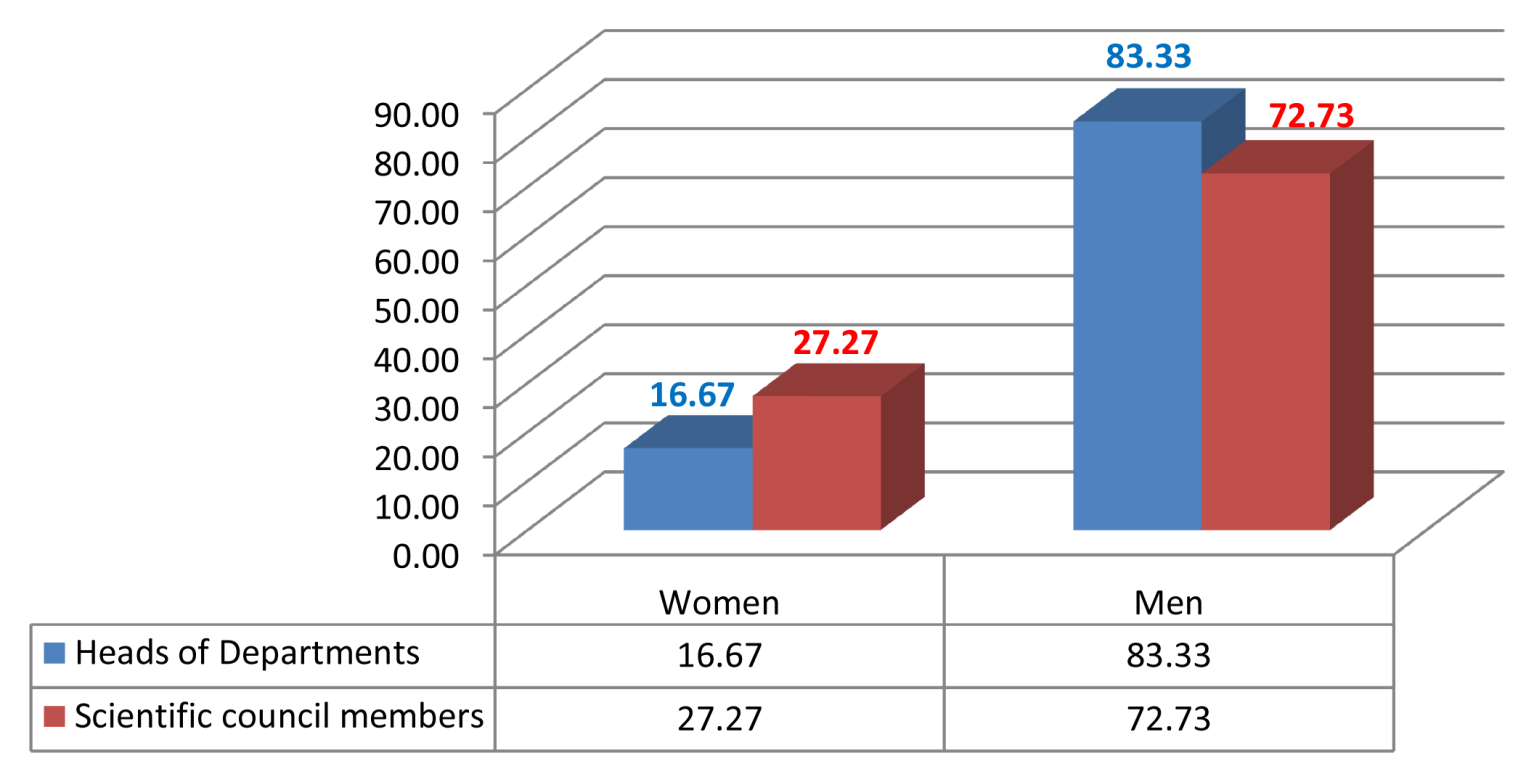
Leadership positions at the Faculty of Arts and Human Sciences of Sousse.

**Figure 5.**
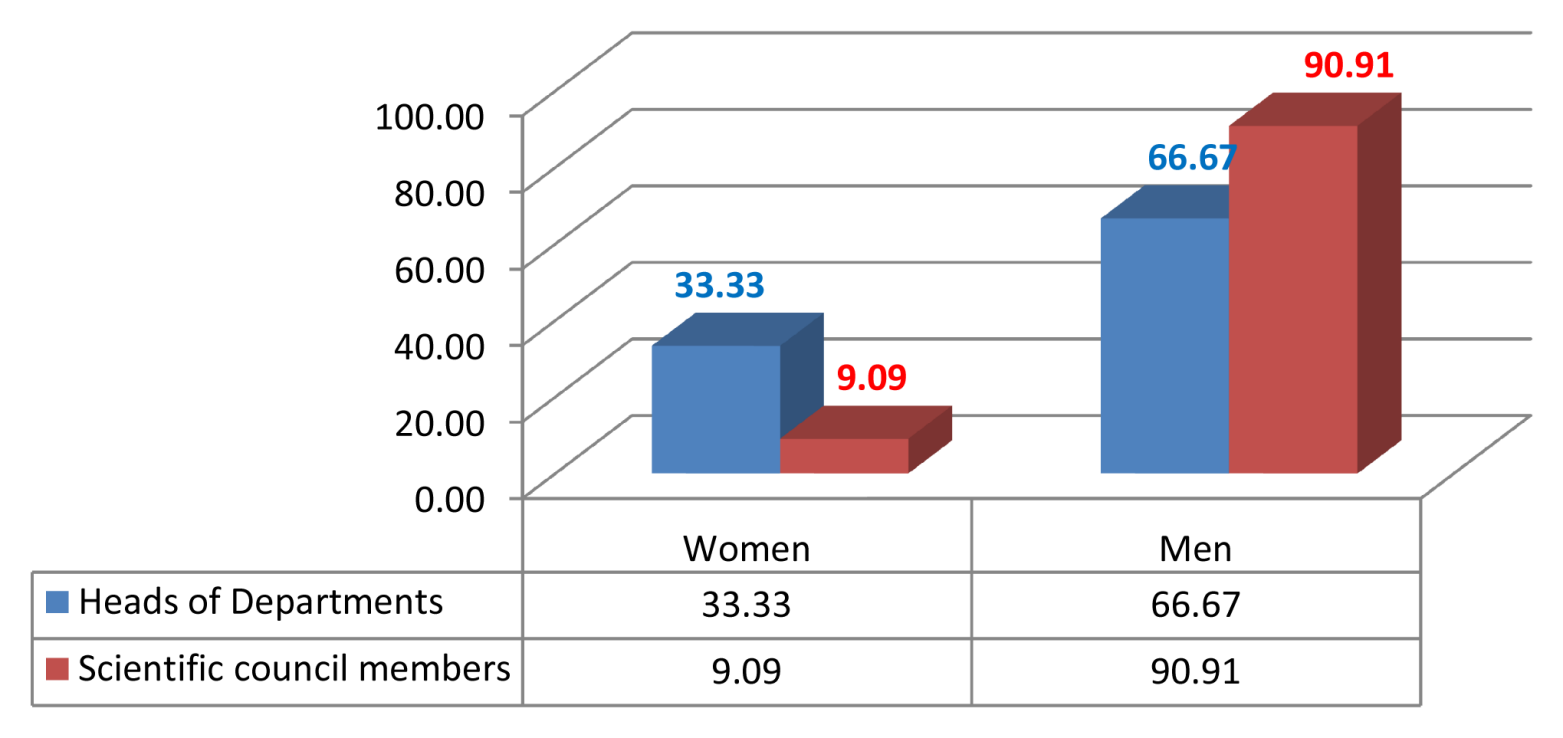
Leadership positions at the National Engineering School of Sousse.

Given the current research area, we measured male/female inequality by calculating the number of men and women who were in leadership positions such as the president of the university or rector, dean, vice dean, HEI director, head of department, member of the scientific university council and/or member of the scientific institutional council ( Figure 2, Figure 3, Figure 4, Figure 5).

We analysed the data and the discrepancies between the percentages of women and men in both institutions; a fact that indisputably serves to enforce the argument of established gender inequality in HEIs. With gender equity, impartiality is an asset. So, of the 171 staff members in FAHSS ( Figure 2), 78 are women and 93 are men (45,61% and 54,38% respectively). By the same token, of the 83 staff members at ENISO ( Figure 3), 24 are women and 59 are men (28,9% and 71,1% respectively). We see a big difference between the two institutions, with a greater gender balance within humanities and STEM is far more unbalanced in terms of the representation of women.

Despite the minute differences in terms of the diverse disciplines in each higher education institution, the results regarding gender inequality in academic promotion are almost the same. At the FAHSS, the number of grade A women who attained full professor is 2 compared to 19 male peers ( Figure 2). At ENISO, it looks the same because there is 1 woman compared to 18 male colleagues in grade A ( Figure 3). As to the lower grade of associate professors, while there are 9 women (42.85%) compared to 12 men (57.14%) at FAHSS, there are only 2 women out of the 5 associate professors at ENISO (40%).

Noticeably, gender inequality regarding leadership positions is an issue in consistency with the low representation of women in academic promotion, such as full professors or associate professors. Therefore, women are behind in the attainment of high profile careers at FAHSS and ENISO at Sousse University ( Figure 4, Figure 5).

### Underrepresentation of women in humanities (FAHSS)

While women represent just over half (50.96%) of assistant professors and are beyond parity (48.04%) they do not progress to the status of associate professor and/or full professor in the same period of time as their male counterparts do in the same institution. In other words, the increasing number of women in junior teaching positions does not systematically imply a growth in the number of women in senior positions. While the rising number of men’s senior teaching positions as associate professor is 12 out of 21 (57.14%), the number of women is 9 out of 21 (42.85%), which is still lower than parity. The number of men full professors is 19 out of 21 (90.47%), while women represent only 2 out of 21 (9.52%). The statistics as shown in the graphics are bewildering but true to the FAHSS data, which accounts for the legitimacy of the controversial issue of academic promotions that this study labels as unjust.

Being a preliminary requisite to leadership positions, academic promotion is more advantageous for men than women, especially in the FAHSS. For example, in the Arabic department the percentage of men who reached positions within the top-grade A (full professors) compared with women means that more men full professors were candidates for deanship than women. The recent 2020 electoral results, as the chart displays, are a testimony to this. ( Table 1).

**Table 1.** Higher education institutions electoral results finalised December 2020, Sousse University.

Institutions	Head of Dpt: Male	Head of Dpt: Female	Scientific Board Grade A: Male	Scientific Board Grade A: Female	Remarks	Scientific council Grade B: Male	Scientific council Grade B: Female
1. Faculty of medicine	3/8	5/8	6/7	1/7	Dean: male	0/6	6/6
2. Faculty of Law and Political sciences	1/2	1/2	3/6	3/6	Dean: female	3/5	2/5
**3. Faculty of Arts & Human Sciences**	5/6	1/6	4/6	2/6	Dean: male	4/5	1/5
4. Higher Institute of management	1/4	3/4	2/5	3/5	Director: male	2/2	2/2
5. Higher Institute of App. Sciences & Technology	4/4	0/4	4/5	1/5	Director: male	2/5	3/5
6. Higher Institute of Music	1/1	0/1	--	--	Director: male	4/5	1/5
7. Higher Institute of Fine Arts	2/3	1/3	3/5	2/5	Director: male	3/5	2/5
*8*. Higher Institute of Transport and Logistics	3/3	0/3	3/3	0/3	Director: male	3/5	2/5
9. Higher Institute of Computer and Com Technics	1/2	1/2	4/5	1/5	Director: male	4/4	0/4
10. Higher Institute of Agricultural Sciences	1/4	3/4	2/6	3/6	Director: male	3/5	2/5
11. Higher Institute of Finance and Taxation	1/2	1/2	2/2	0/2	Director: male	1/4	3/4
12. High School of Commerce	1/2	1/2	2/5	3/5	Director: female	2/4	2/4
**13. National Engineering School of Sousse**	2/3	1/3	6/6	0/6	Director: male	4/5	1/5
14. Higher Institute of Sciences & Technology	3/3	0/3	5/6	1/6	Director: male	3/5	2/5
15. Faculty of Economic Sciences and Technology	1/3	2/3	4/4	0/4	Dean: male	4/4	0/4

As to the academic title associate professor, which is preliminary to attaining full professor, the percentage of men remains higher than their women peers. Equally in the History department, the percentage of men who reached grade A, i.e. full professor, is 100% compared with just one woman who is an associate professor. As to the academic title associate professor, similarly, in the Arabic department there have been only men associate professors previously, except this year (2020) four women reached the title associate professor. The English department is theoretically expected to comprise more open-minded teaching teams (it is the first department initiating gender courses since 1998–1999) than the staff of the other four departments, without including the recently established department of Anthropology and African Studies. However, for two decades, the subsequent heads of the department have been males. The only one woman who reached the level full professor in the last decade of her career.

### Underrepresentation of women in academia in the Engineering School (ENISO)

Numbers and percentages of women and men in the different academic positions and leadership management highlight the index of dissimilarity and, similarly to FAHSS, the systematic gender inequality that matters on many levels in this paper. Although ENISO prides itself in the increasing number of women accessing the elitist STEM institution, this access has shown no evidence of promoting GE in promotion or in leadership positions ( Figure 4).

## Discussion

Gender equality has captivated the attention of the HE staffin Tunisia. In humanities, the study of gender has been looked down on as below their elitist concerns. However, the exploration of this issue in this paper will reveal unspoken or latent bewildering gender issues in the field of higher education. The significance of gender equality has become more crucial than ever in a teaching milieu based on gender segregation.

Although the number of women in leadership positions started increasing, it remains very low. As mentioned in the chart of the electoral results, one woman elected in the management position of dean at the Faculty of Law and Political Sciences compared to other three men deans at the FAHSS, Finance, Economics and Medicine. Also, while one woman has been elected director at the High School of Commerce, there are 12 men directors of other HEIs. Finally, one more woman full professor was appointed manager of the paramedical school.

In line with some conclusions in the European research about gender inequality in higher education institutions, the numbers of women in FAHSS and ENISO are improving, albeit slowly. Equally, according to the latest European Commission ‘SHE’ figures handbook, in 2012 only 33 % of European researchers were women and this number becomes lower in male-dominated fields ^3^.

Tracing the history of women in academia, there is evidence of their under-representation within decision-making-positions starting at the level of managers at higher education institutions, or as heads of departments. Even when it is a position held by appointment, existing male networks never or rarely choose a woman leader. These findings are disappointing as, even after the so-called democratization process after the Arab Spring, the percentage of female instructors and researchers holding PhD degrees increased, but without being equaled in academic promotion and/or leadership positions.

The 2020 institutional elections at Sousse University start with the candidates to become heads of departments, then the candidates to become members of the institutional scientific council, the dean or the director, and the candidates of the members of the university council till we reach the last and top leadership position of the president of the university or rector. Elections take one month from the electoral campaign results, that support the evidence of the results of this study.

Results from the 2020 elections point to similar perceptions of gender inequality in leadership positions, as only 10% of the directors of institutions are women. There is an increasing number of women in HEIs in institutional and university councils, with about 20% of women sharing decision-making positions or responsibility posts.

Because the dominant patriarchal university culture determines the institutional set of norms, the low representation of women in grade A positions at both leading institutions mirrors the impact of the patriarchy-oriented culture at higher education levels. Therefore, the act of interrogating the deep-rooted gender inequality in HEIs is a pathway to deconstruct this, so as to develop the structure of gender relations along with their bearing on academic promotion and leadership policies at Sousse University.

Regarding the results, the far-reaching effect of patriarchal culture is sharply observed which necessitates revisiting the understanding of gender balance, which is needed due to the fast-developing globalization and cyberspace networks. In fact, the profound examination of the current cultural standards with regard to women’s academic promotion as well as their access to management positions in HEIs unveils a great impact of the patriarchal society especially the prevailing domestic ideology that ties women to the theory of biology as destiny. The latter has enforced structural barriers that the current project at Sousse University aims to end it.

### Surprises

When the current study is *a première*, that means we do not know a lot in terms of exact facts and numbers unless charts, graphs and statistics are provided, the presuppositions turn out to be mere fallacies. For example, we thought beforehand that women as engineers at ENISO are able to pursue a grade A position over a shorter period of time than men. However, their low number compared to their male peers is the same as in the FAHSS, with one exception that could not be a rule. Many women have not yet reached grade A among the institutional staff members, i.e. associate professors or full professors, although many of them assume the role and tasks of both academic titles, including presenting lectures and supervising MA theses and doctoral dissertations.

What is mostly surprising is that many college women have internalised gender inequality as a given or as a biological determinant, which endorses sexist discrimination. Built upon this finding, female teachers tend to normalise the belated academic promotion while placidly admitting the structure of leadership policies. In the light of this deduction, we understand some women’s rejection of the deconstruction of gender stereotypes; there are few exceptions. After 2011, the spirit of some scholars at HEIs, where an anti-stereotypical, modernised and innovative spirit is expected to prevail, conforms with gender distinction beliefs that obstruct GE development strategies.

### What is most striking about the tables, figures and illustrations?

Upon reading the facts in this study, there is a wide sense of salient discomfort, albeit unsaid, within the HEI community in proclaiming modernization and democratization of e-Learning or distance learning, but thwarting the apparatus of progress to achieve equal rights to attain leadership positions in academic settings. What is bewildering is that the numbers and percentages that we observe in tables, graphs, and statistics showing the increasing number of women in HEIs do not equal the percentages of women in management positions, which are still below expectations.

### The family life and cultural dimensions

Although GE is a means to advance the humanistic dimension in the academic community, gender inequality is partly an outcome of cultural factors, deep-seated patriarchy-oriented norms affecting the culture at HEIs.

Given the results, which show evidence of sexual discrimination and enhance gender inequality, we deduce that the spirit of uncultivated academic community impacts on leadership and decision-making positions. The latter have so far been framed within male-biased networking that has been a challenging cultural dimension at Sousse University.

The patriarchy-oriented culture has been internalised by both genders to slow down or even obstruct gender balance development as regards academic promotion. Relevant to implicit person theory principles ^10^, women are opinionated about women in authority, which is an outcome of a woman’s belief in herself. Gender bias should be moderated to leave room for self-esteem, especially among women, to develop their status from within. That’s to say a redefinition and a reconceptualization of a woman as to her self-esteem is a must, to mentally and cognitively see herself worthy of the status of leader at HEIs.

**Figure 6.**
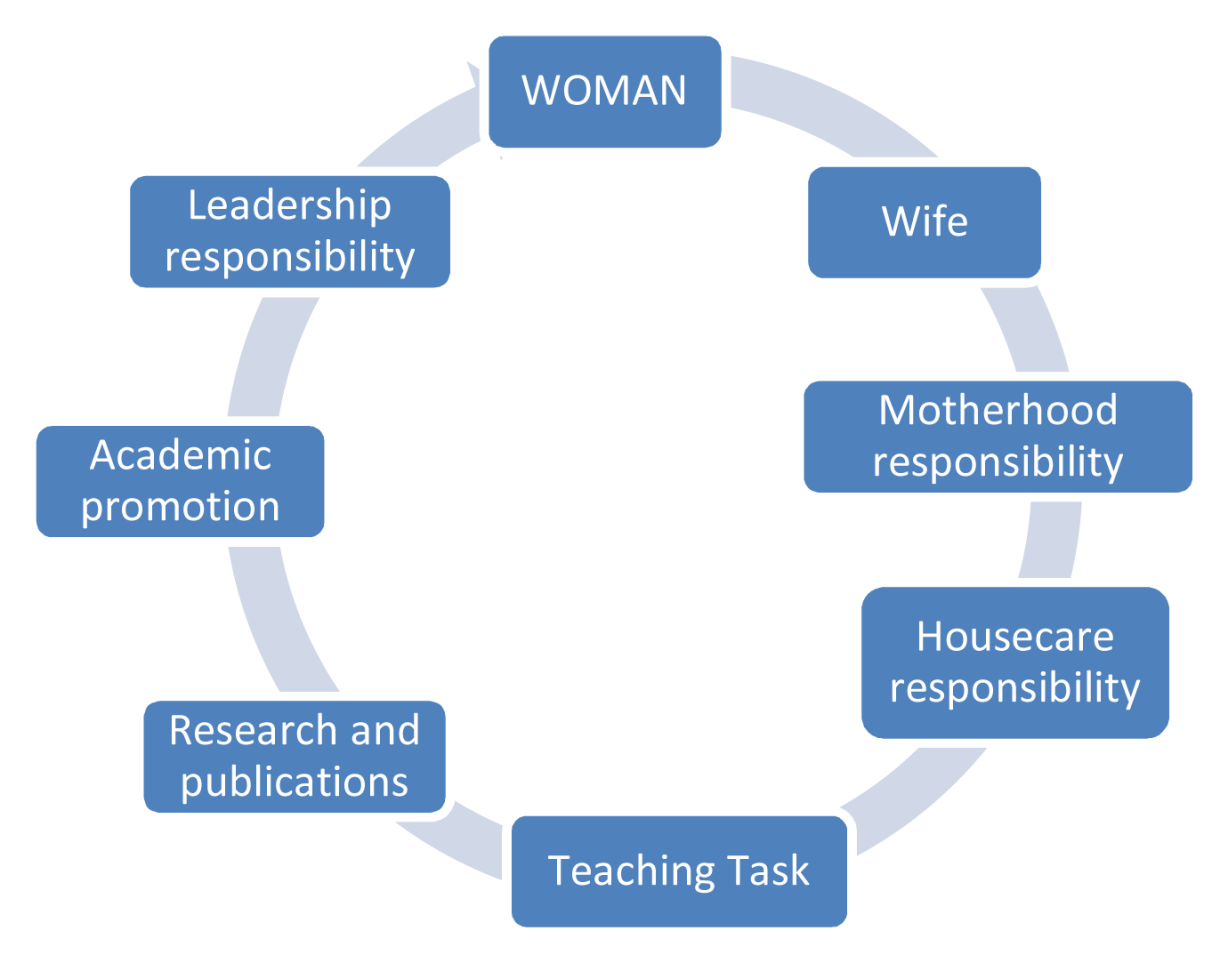
Academic and family duties of female academics.

If women, like men, are not cognisant of the benefits of GE as beneficial on the productivity level, gender inequality rises. Therefore, when a woman is a wife and mother responsible for childbearing and house care tasks, her research combined with teaching task hinder her academic promotion, that remains in the lowest order of her professional achievements ( Figure 6).

With regard to Figure 6, we can see the ‘glass ceiling’ metaphor epitomised in the different tasks that are combined together under one heading, WOMAN, in bold and capital letters. What patriarchy-oriented cultures ignore is that most women who ‘silently’ sacrifice to keep the domestic sphere safe and thriving are not short of academic potential and leadership competencies.

In the same line of thought, we cite Bernie Grummell *et al*. (2009) ^11^ who approves of the opinion that ‘understanding how the care ceiling operates is crucial for understanding why women do not occupy senior managerial positions within new managerial regimes in higher education’.

Women presidents are less likely than male presidents to be married or have children and are more likely to have altered their careers for the equilibrium of their family. This situation remains thought-provoking for advocates of gender equality as a requisite of human rights principles in favour of promoting social justice. Nevertheless, for many women, family responsibility matters more than professional development. According to the same study ^11^ since higher education is “subject to performance measurement and rankings,” that apply more to men than women, within the case study of Irish higher education, we see this as applicable to Tunisian HEIs.

Male colleagues in HEIs preserve a majority in academic responsibilities, such as coordination, supervision and training, which systematically makes the same names eligible for institutional leadership positions. The percentages of the recent 2020 elections at Sousse University is slightly moving the needle above the stereotypes, which the diagram above endorses. Despite the idea that it is to the benefit of higher education institutions to speed up an advanced gender equality developing process, the numbers do not serve as confirmation of this.

The issue of gender imbalance remains persistent which mirrors wider patterns of discrimination and devaluation of women’s potential as full human beings.

Therefore, GE involves three fundamental tasks:

(1)   creating a culture that boosts women’s potentials to see promotion as an obligation and not a choice;

(2)   shaping cultural norms within the HE milieu to brainstorm women and men of the fact that promotion is an obligation and not a choice; and

(3)   creating a network ensuring interconnection within an academic community sharing common gender-unbiased interests.

## Next steps

This paper makes its main claim about gender inequality in academic settings, with a focus on the teachers’ progression in their professional career. So, to be able to ‘move the needle forward’ towards gender equilibrium, a combination of efforts is needed, joining GES at the micro, meso and the macro levels to reduce the domination of deep-seated male dominated institutional practices that have gone through the Butlerian “normativity” process ^12^.

Our recommendations are the following:

▪   Bear in mind that bridging the gender gap at HEIs requires substantial promotion of the participation of women in research projects and academic network of publications.

▪   Increase mobility across HEIs in the country and abroad to help develop the mindsets of academic staff towards a better gender balance in academic promotion and leadership positions.

▪   Insert gender identity construction in the content of the course and organise tutorials about enhancing female self-esteem to further boost leadership skills among women in order to interact with their male peers.

▪   Create a Gender Cell at Sousse University to endorse egalitarianism between sexes and enhance the Gender Equality dimension. This structure will enable both academic and administrative staffs to enjoy a healthy work environment leading to collegiate professional evolution.

### What is implied or proposed for future study? Women/WeMen Council move the needle

Coordinating with the EU TARGET H2020 project to investigate deep-seated gender issues from a wide spectrum of interdisciplinary perspectives and approaches, in humanities and STEM institutions, we propose the following:

▪   Design a teaching curriculum built upon a vision of promoting gender equality in every aspect of academia and, therefore, integrate the gender dimension in research and innovation curriculum.

▪   Integrate the gender dimension in research and teaching content. By highlighting the structural barriers, this paper contends to show a thread of light by the establishment of a Master’s Program about Women/Gender Studies and most important is the implementation of gender equity and gender equality in different teaching modules at the FAHSS among other HEIs.

▪   Launch a Women/WeMen Council, a pioneering enterprise, at Sousse University, to boost gender parity and, simultaneously, to help HE intellectual scholars revisit their view of GE to see it from within, and to better grasp it and make it intrinstic in their daily dealings.

▪   To organise workshops, mini-conferences, study days and info days entitled ‘gender equality as a key dimension in daily life at higher education institutions’ to encourage women teachers to become knowledgeable of their roles as human beings rather than female beings.

To this effect, the current study chases the vision of framing GES to develop a pattern or a guide of gendered policies in HEIs. A Women/WeMen Council represents a embryonic vision of a wide scope or speculum of strategies a world model for gender equilibrium; an outcome of stepping up and ‘moving the needle’.

### How far can RMEI strategies help promote GE policies?

By unveiling gender-biased discrimination, this paper opens the floor for further exploration of the dynamics of power relations and gender inequalities that the whole organic sytem, at the micro and macro levels, keeps perpetuating, either consciously or unconsciously.

### Perspective on academic promotion in higher education in Tunisia

Understanding the gap between gender equality and male dominance in decision-making positions involves many challenges. Informed by institutional analysis and feminist institutionalist scholarship, this study explores the relationship between academic promotion and gender equality.

By using the indicator of promotion related to gender equality, we opt for a scrutiny of culture-biased norms that are central to institutional change. There is a consensus demonstrating that institutional analyses improve when gender dynamics are incorporated. Showing the gendering of power relations highlights power in institutional change in new ways, improving understandings of why institutional change rarely happens as intended by institutions.

## Conclusions

This study is supported by the EU TARGET project, that extends beyond the European borderlines to establish GES in coordination with ENISO’s Gender Equality Center at Sousse University, created in 2018 with the support of RMEI. It is aiming to introduce a process of transformation at higher education institutions in Tunisia and other Mediterranean countries.

Under the framework of the TARGET project and in the same vein of thought of tackling gender inequality as a prominent issue, the developed and adopted Gender Equality Policy Statement (2018) by the RMEI was also shared and adopted by ENISO that has recently engaged the FAHSS by virtue of this research, for which an objective is to promote mechanisms and policies of egalitarianism in higher education institutions.

The current GE statement gained significance due to the status quo of gender inequality in Tunisian HEIs, evokings the need to investigate the prevalent issues of academic promotion and leadership and the commitment of university’s leaders to SDGs and GE.

In line with the main goal of the TARGET H2020 project, this paper attempts to contribute to the advancement of gender equality in Research and Innovation by taking into consideration the first dimension, Gender-related institutional barriers to career development, and the second one leadership positions. However, when it comes to the third dimension, implementation of Research and Innovation in the curricula content, we have launched it in teaching and research work.

By highlighting the structural barriers, this paper contends to show a thread of light by the creation of new content, inspired by the TARGET project, related to Licenses and Master’s Program about Women and Gender Studies as well as the implementation of gender equity and gender equality concept in different teaching modules at Sousse University.

What mostly triggered our desire to tackle gender inequality in this study is our belief in the necessity of developing a process to change the power-relations structure that fosters gender equality, explicitly or implicitly, in academic institutions. Therefore, this paper examined deep-seated gender inequality in academic promotion and leadership in two leading institutions at Sousse University (Tunisia): using FAHSS and to a lesser degree ENISO as case studies.

Therefore, the study is interdisciplinary as it addresses gender issues with a focus on gender inequality in academia in terms of promotion and leadership positions. By unveiling gender-biased discrimination, it opened the door for further exploration of the dynamics of power relations and gender inequalities that either consciously or unconsciously affects the whole organic system, at the micro and macro levels, proposing some fundamental steps in advancing gender balance in Tunisian Institutions. To this effect, the current study chases the vision of framing GES to develop a pattern or a guide of gendered policies in HEIs.

While this paper highlights the critical situation of the underrepresentation of women in HEIs in Tunisia, it raises attention for the need of improvement to help increase the number of women in senior academic roles and leadership positions and provides recommendations.

The “moving the needle” metaphor is necessary in order to push forward academic staff’s commitment to developing a program of gender equality process, focusing on gender equality in engineering institutions and highlighting the need for investigation methodologies to endorse equality strategies towards the establishment of higher educational networks.

Finally, this case study would, for many objective reasons, apply to other higher education institutions in Tunisian universities, in other Mediterranean countries and worldwide.

## Data availability

DANS: FAHSS-ENISO Gendered DATA

https://doi.org/10.17026/dans-xdn-9g3a ^13^

This project contains the following underlying data:

- FAHSS Gendered Open Data.csv (It comprises the total number of the academic staff at FAHSS including both genders in both grades A and B with minute details about the exact number of each gender within each category, added to leadership positions)
- ENISO Gendered Open Data.csv (It comprises the total number of the academic staff at ENISO including both genders in both grades A and B with minute details about the exact number of each gender within each category, added to leadership positions)

Data are available under the terms of the Creative Commons Zero "No rights reserved" data waiver (CC0 1.0 Public domain dedication).
