# Supplementary material for: Academic promotion and leadership: ‘moving the needle’ for the enhancement of gender equality in Tunisian higher education institutional members of the RMEI network following the TARGET framework
Source: Open Res Eur. 2021 May 13;1:14. Originally published 2021 Mar 24. [Version 2] doi: 10.12688/openreseurope.13217.2 (PMC10445811; doi:10.12688/openreseurope.13217.2)
Supplement: Peer review history

# Peer review: Reviewer response for version 2

**DOI:** 10.21956/openreseurope.14678.r26883

**Published:** 2021-06-01

**Authors:** Angela Wroblewski

**Affiliations:** 1 Institute for Advanced Studies IHS, Vienna, Austria

The authors further developed the paper by focusing to a comprehensive gender equality concept and by referring to it consistently in the analysis as well as discussion. The paper represents an important contribution to the emerging gender equality discourse in Tunisian academia.

If any, are all the source data and materials underlying the results available?

Yes

Is the work original in terms of material and argument?

Yes

Is the work clearly and cogently presented?

Yes

Is the argument persuasive and supported by evidence?

Partly

Does the research article contribute to the cultural, historical, social understanding of the field?

Yes

Does it sufficiently engage with relevant methodologies and secondary literature on the topic?

Partly

Reviewer Expertise:

Gender equality in R&I, structural change, evaluation of gender equality policies.

I confirm that I have read this submission and believe that I have an appropriate level of expertise to confirm that it is of an acceptable scientific standard.

# Peer review: Reviewer response for version 1

**DOI:** 10.21956/openreseurope.14287.r26574

**Published:** 2021-04-12

**Authors:** Angela Wroblewski

**Affiliations:** 1 Institute for Advanced Studies IHS, Vienna, Austria

The paper provides a first analysis of women’s representation in academic positions in Tunisia and therefore an important starting point for a gender equality discourse in Tunisian academia. Based on data of two faculties of Sousse University the current gender composition of academic positions and decision-making positions is analysed. In a second step reasons for gender imbalances are discussed and in a third step measures to support a gender balanced representation are recommended.

 As already mentioned, the paper could provide a starting point for a gender equality discourse in Tunisian academia. To support such a gender equality discourse it would be helpful to provide a definition of gender equality. The authors do not explicitly define gender equality but refer to the framework provided by the EU-funded structural change project TARGET. TARGET – like all structural change projects – is based on a comprehensive gender equality construct which addresses women’s representation, the abolishment of structural barriers for women’s careers and the integration of the gender dimension in research and teaching content. The paper focuses on the first of the three gender equality dimensions – women’s representation. To avoid the impression that gender equality is reduced to one dimension, the underlying gender equality concept should be expounded.

 The discussion section as well as the proposed next steps provide food for thought for a national discourse about gender equality in R&I. Even though measures like the Women/WeMen Council at Sousse University or the integration of the gender dimension in curricula carry enormous potential to contribute to awareness raising regarding gender equality issues, the main focus is on women and not on structures.

 To continue the important work stated with this first paper I suggest to expand the future analysis by focusing on structures and processes. However, as a first step the analysis presented should be enriched by providing more information about the context (e.g. share of women among students) as well as by explicating the underlying gender equality concept.

If any, are all the source data and materials underlying the results available?

Yes

Is the work original in terms of material and argument?

Yes

Is the work clearly and cogently presented?

Yes

Is the argument persuasive and supported by evidence?

Partly

Does the research article contribute to the cultural, historical, social understanding of the field?

Yes

Does it sufficiently engage with relevant methodologies and secondary literature on the topic?

Partly

Reviewer Expertise:

Gender equality in R&I, structural change, evaluation of gender equality policies.

I confirm that I have read this submission and believe that I have an appropriate level of expertise to confirm that it is of an acceptable scientific standard, however I have significant reservations, as outlined above.

# Response

**Published:** 2021-05-13

**Authors:** MONCEF GHISS

**Affiliations:** National Engineering School of Sousse, Tunisia

**Comment** 1: The paper provides a first analysis of women’s representation in academic positions in Tunisia and therefore an important starting point for a gender equality discourse in Tunisian academia. Based on data of two faculties of Sousse University the current gender composition of academic positions and decision-making positions is analyzed. In a second step reasons for gender imbalances are discussed and in a third step measures to support a gender balanced representation are recommended. As already mentioned, the paper could provide a starting point for a gender equality discourse in Tunisian academia. To support such a gender equality discourse it would be helpful to provide a definition of gender equality. The authors do not explicitly define gender equality but refer to the framework provided by the EU-funded structural change project TARGET. TARGET – like all structural change projects – is based on a comprehensive gender equality construct which addresses women’s representation, the abolishment of structural barriers for women’s careers and the integration of the gender dimension in research and teaching content. 

**Reply** 1: As rightly suggested, we have added in introduction this definition of gender equality:   Institutional Gender equality implies that the number of women reaching high profile career in HEIs must be the same as that of men. In line with this definition, access to management opportunities, Research and Innovation project participation and other empowering positions, regardless of gender, have become a must. To achieve these fair objectives, a structural change must be implemented within university culture. As TARGET H2020 project boosts a structural change, we have adopted three dimensions of gender equality as outlined in this collaborative research project. First, the gender balance by examining gender-based issues and investigating the real gap hindering equality. Second, the abolishment of barriers for professional careers development of women by the establishment of Gender Cell at Sousse University and the ENISO’s Center of Equity impacting the different institutions to propagate and disseminate gender norms of equality. Third, the integration of the gender dimension in research content by the establishment of a Master’s Program about Women/Gender Studies as well as the implementation of gender equity and gender equality in different teaching modules at the Faculty of Arts and Human Sciences among other HEIs.

**Comment** 2: The paper focuses on the first of the three gender equality dimensions – women’s representation. To avoid the impression that gender equality is reduced to one dimension, the underlying gender equality concept should be expounded.

**Reply** 2: As rightly suggested, we have added this paragraph in conclusion. In line with the main goal of the TARGET H2020 project, this paper attempts to contribute to the advancement of gender equality in Research and Innovation by taking into consideration the first dimension, Gender-related institutional barriers to career development, and the second one leadership positions. However, when it comes to the third dimension, implementation of Research and Innovation in the curricula content, we have launched it in teaching and research work.   As suggested by the reviewer we have clarified the other two dimensions of gender equality, (2) the abolishment of structural barriers for women’s careers and (3) and the integration of the gender dimension in research and teaching content by adding these two paragraphs    (2) the abolishment of structural barriers for women’s careers (Discussion) The profound examination of the current cultural standards with regard to women’s academic promotion as well as their access to management positions in HEIs unveils a great impact of the patriarchal society especially the prevailing domestic ideology that ties women to the theory of biology as destiny. The latter has enforced structural barriers that the current project at Sousse University aims to end it.    (3) the integration of the gender dimension in research and teaching content. (Conclusion)  By highlighting the structural barriers, this paper contends to show a thread of light by a creation of new content, inspired by the TARGET project, related to License’s and Master’s Program about Women/Gender Studies as well as the implementation of gender equity and gender equality concept in the curricula of other HEIs.

**Comment** 3: The discussion section as well as the proposed next steps provide food for thought for a national discourse about gender equality in R&I. Even though measures like the Women/WeMen Council at Sousse University or the integration of the gender dimension in curricula carry enormous potential to contribute to awareness raising regarding gender equality issues, the main focus is on women and not on structure. To continue the important work stated with this first paper I suggest expanding the future analysis by focusing on structures and processes.  However, as a first step the analysis presented should be enriched by providing more information about the context (e.g. share of women among students) as well as by explicating the underlying gender equality concept.

**Reply** 3: We agree with this comment. As suggested by the reviewer we have expanded the future analysis by focusing on structures and processes. We have also provided more information about the context.  Structures (added in section 6 Next step?)  The Establishment of Gender Cell at Sousse university to endorse egalitarianism between sexes and enhance the Gender Equality dimensions. This structure enables both academic and administrative staffs to enjoy a healthy work environment leading to collegiate professional evolution. Processes (added in Conclusion?) the same as comment to reply 2 Integrate the gender dimension in research and teaching content. By highlighting the structural barriers, this paper contends to show a thread of light by the establishment of a Master’s Program about Women/Gender Studies and most important is the implementation of gender equity and gender equality in different teaching modules at the Faculty of Arts and Human Sciences among other HEIs.

# Peer review: Reviewer response for version 1

**DOI:** 10.21956/openreseurope.14287.r26586

**Published:** 2021-04-07

**Authors:** Maria Caprile

**Affiliations:** 1 Notus: Applied Social Research, Barcelona, Spain

The article provides new data on women's underrepresentation in senior career positions and decision-making positions in two Higher Education institutions in Tunisia. Data in both institutions depict a similar picture and provide evidence for gender equality policies supporting women's promotion and counteracting gender prejudices and traditional gender norms - for both women and men.

 The article builds on this to elaborate specific recommendations for these institutions and Higher Education in general in Tunisia. It is an article which provides valuable data, reflection and recommendations to advance gender equality.

 Minor aspects which could be addressed to improve the article:

1. "Another important issue is sexual harassment in Universities. In 2016, the European Institute for Gender Equality published an inspiring comprehensive study in support of the elimination of **sexual distinction**, to ensure gender equity and gender equality in academia".
  The reference to "sexual distinction" is not fully clear. Bibliographical reference is missing. 
2. Women/WeMen: it seems a highly inspiring initiative. It could be further developed.

If any, are all the source data and materials underlying the results available?

Yes

Is the work original in terms of material and argument?

Yes

Is the work clearly and cogently presented?

Yes

Is the argument persuasive and supported by evidence?

Yes

Does the research article contribute to the cultural, historical, social understanding of the field?

Yes

Does it sufficiently engage with relevant methodologies and secondary literature on the topic?

Yes

Reviewer Expertise:

Gender in R&I, employment inequalities.

I confirm that I have read this submission and believe that I have an appropriate level of expertise to confirm that it is of an acceptable scientific standard.
